# Investigating Predictors of Nurses’ Professional Quality of Life and the Role of Cyberbullying: A Cross‐Sectional Study

**DOI:** 10.1155/jonm/7537227

**Published:** 2026-07-24

**Authors:** Fatemeh Malekghasemi, Matin Arabpoor, Sara Mehdizadeh, Morteza Rabiei Vaziri, Mahlagha Dehghan, Parvin Mangolian Shahrbabaki

**Affiliations:** ^1^ MSc Student of Pediatric Nursing, Reproductive and Family Health Research Center, Kerman University of Medical Sciences, Kerman, Iran, kmu.ac.ir; ^2^ MSc Student of Critical Care Nursing, Nursing Research Center, Razi Faculty of Nursing and Midwifery, Kerman University of Medical Sciences, Kerman, Iran, kmu.ac.ir; ^3^ MSc Student of Emergency Nursing, School of Nursing and Midwifery, Shahid Beheshti University of Medical Sciences, Tehran, Iran, sbmu.ac.ir; ^4^ MSc Student of Critical Care Nursing, Nursing Research Center, Kerman University of Medical Sciences, Kerman, Iran, kmu.ac.ir; ^5^ HIV/STI Surveillance Research Center, and WHO Collaborating Center for HIV Surveillance, Institute for Futures Studies in Health, Kerman University of Medical Sciences, Kerman, Iran, kmu.ac.ir; ^6^ Nursing Research Center, Kerman University of Medical Science, Kerman, Iran, kmu.ac.ir

**Keywords:** burnout, cyberbullying, nurses’ compassion satisfaction, secondary traumatic stress

## Abstract

**Background:**

Digital technology has brought many advantages to healthcare, but it has also given rise to a new form of worker abuse called “cyberbullying.” This type of bullying can happen anytime and can greatly harm workers’ mental health. Since “professional quality of life” is vital for how well the healthcare system functions and there is not much research on how this connects to online bullying in Iranian nursing, it is important to look closer at this relationship. This study aimed to assess the association between cyberbullying and professional quality of life among nurses in Southeast Iran.

**Materials and Methods:**

This research utilized a descriptive cross‐sectional design and recruited 335 nurses from Kerman University of Medical Sciences affiliated hospitals during 2025. Subjects were enrolled using a convenience sampling approach, and data were collected using the Menesini Cyberbullying Questionnaire and the Professional Quality of Life Scale (ProQOL). Data were analyzed using SPSS Version 24 and descriptive statistics, independent *t*‐tests, analysis of variance, spearman correlation coefficients, and stepwise multivariate regression, with a significance level of less than 0.05.

**Results:**

Data analysis indicated a low prevalence of cyberbullying among nurses. The mean scores of compassion satisfaction, burnout, and secondary traumatic stress were 34.71 ± 5.47, 16.03 ± 3.92, and 31.63 ± 4.90, respectively, all of which fell within the moderate range. Inferential findings indicated a significant positive correlation between cyberbullying and compassion fatigue, burnout, and secondary traumatic stress. Furthermore, cyberbullying emerged as a significant positive predictor across all three dimensions of professional quality of life in regression analyses. Additionally, key demographic and occupational factors including educational degree, shift type, employment status, gender, and marital status emerged as significant predictors within their respective models.

**Conclusions:**

The findings indicated that cyberbullying was generally low and showed significant statistical associations with compassion fatigue in the analyses. Targeted interventions to enhance nurses’ professional quality of life should be prioritized in healthcare settings. Furthermore, longitudinal and interventional studies are needed to better elucidate the temporal and causal relationships among these variables.

**Implications for Nursing Management:**

Nurse administrators should adopt preventive strategies addressing workplace cyberbullying and compassion fatigue to enhance nurses’ professional quality of life.

## 1. Introduction

The clinical and professional environment of nursing is intertwined with numerous and complex challenges that profoundly affect the mental health and occupational efficiency of healthcare providers. Scientific evidence shows that factors such as long hours of service delivery, environmental stresses, and the imbalance between reward and effort lay the groundwork for decreased job satisfaction and the occurrence of burnout in the long term [1–3]. Among the wide range of these challenges, “workplace bullying” has been identified as a harmful and serious threatening component for the well‐being of nurses [4, 5], which, in recent decades and parallel to the digitalization of health system processes, has changed form and emerged in a new format called “cyberbullying.” This phenomenon possesses dimensions, characteristics, and consequences distinct from traditional bullying [6, 7].

Cyberbullying in the workplace context refers to the intentional and repeated performance of negative behaviors through digital communication tools with intentions such as harassment, intimidation, humiliation, or harming colleagues, which endangers their emotional well‐being [6, 8, 9]. Today, this phenomenon has become a challenge for human resource management in the health system [8]. Cyberbullying has a “borderless” nature and can target the victim 24 h a day, while traditional bullying is usually limited to the hours of presence in the ward. This feature transforms the stressor from an acute occupational factor into a chronic psychological conflict that follows the individual even into the haven of their home [6, 10]. Additionally, the high speed of dissemination and the public nature of virtual space can create unique anxiety and distress in victims that severely destroy their self‐esteem and job performance [5, 6].

According to recent systematic reviews, in a study by Zhang et al., the prevalence of cyberbullying among healthcare workers was reported between 1.5% and 46.6%, and specifically in nurses, the experience of digital incivility or cyberbullying is estimated to be up to 36.8% [11]. In a study based in New Zealand, D’Souza et al. demonstrated that cyberbullying often occurs alongside traditional bullying, which leads to more extensive pathology in victims [6]. Other global statistics, including an 8% prevalence in South Korea [12], 30.1% among nursing students in Jordan [13], and 34.7% in Nepal [14], attest to the pervasiveness of this problem.

According to studies, the consequences of continuous exposure to digital violence have gone beyond individual harms, and other dimensions of it in work environments must be investigated [4]. The destructive effects of cyberbullying in clinical environments are not limited to human resources but also entail broad organizational consequences, including increased absenteeism and decreased quality of care [2, 15, 16]. Studies have reported that cyberbullying is recognized as a strong predictor for increasing “turnover intention,” which threatens the stability of the workforce [11, 17]. It can be associated with decreased patient safety and increased nursing errors in the workplace [15]. These reports highlight the importance of a deeper investigation into this phenomenon in healthcare environments and underscore the need for a thorough examination of its relationship with key dimensions, such as professional quality of life.

Professional quality of life is a comprehensive construct that explains an individual’s feelings toward their role as a caregiver and has two main dimensions: positive and negative [18]. The positive dimension of this construct is called “compassion satisfaction (CS),” which refers to the satisfaction derived from effectively helping patients [2, 19]. Nurses who report high levels of CS are more likely to establish empathetic relationships with patients. These nurses provide holistic care, which includes emotional and spiritual support [20]. Scientific evidence shows that CS is strongly correlated with active participation in patient safety activities, meaning that the more a nurse enjoys their role as a helper, the more accurate they are and the more committed they are to safety protocols, and consequently, patient treatment outcomes improve [21, 22].

In contrast, the negative dimension is known as “compassion fatigue (CF),” which itself includes two components: “burnout” resulting from environmental factors and chronic stresses and “secondary traumatic stress” (STS) arising from immediate contact with the traumatic experiences and suffering of those receiving care [2, 19]. Burnout is considered a threat to patient safety and the sustainability of health systems, stemming from chronic work‐related stress [23, 24]. Nurses experiencing emotional fatigue show reduced empathy and diminished ability to communicate with patients [25], increasing the risk of clinical errors and adverse events [26].

STS encompasses the psychological pressure experienced when helping or seeking to help the injured, resulting from indirect exposure to patient‐related trauma [27]. This phenomenon has a high prevalence, especially among nurses working in emergency services, intensive care units (ICUs), and oncology units [26]. This occurrence is associated with decreased nurse interest in the patient, reduced job efficiency, and increased errors [4]. Studies in this field show that nurses globally experience moderate to high levels of CF, and this situation is reported to be more acute in Asian regions [28, 29]. Therefore, CF is not only an individual problem but also a costly factor for the treatment system and a threat to public health [16, 28].

Studies indicate that a positive work place environment is positively associated with CS [30, 31]. Despite the vital importance of this issue, research indicates significant gaps in understanding this phenomenon [11]. Most previous studies have focused on traditional forms of bullying [4, 5, 7, 15, 16, 32, 33], and research that comprehensively examines the association between cyberbullying and dimensions of professional quality of life (positive and negative) is limited [4–6]. To date, limited evidence has addressed the association between cyberbullying and all dimensions of professional quality of life, including CS, burnout, and CF, among nurses while considering demographic characteristics. Moreover, cultural determinants significantly influence the perception and incidence of bullying, highlighting a critical need for indigenous research within diverse cultural frameworks. In Iran, despite numerous reports of challenges in the nursing work environment, research that directly and empirically investigates the relationship between cyberbullying and dimensions of professional quality of life has not been conducted.

Considering the widespread usage of social media and the high incidence of workplace violence, this study addresses a critical gap in the literature. This research seeks to determine the prevalence of cyberbullying experienced by nurses in Southeast Iran; investigate the associations between cyberbullying and the three dimensions of professional quality of life—namely, CS, burnout, and STS; and explore demographic and occupational variables that predict outcomes related to professional quality of life.

## 2. Methodology

### 2.1. Study Design

A multicenter, descriptive, cross‐sectional study was used.

### 2.2. Setting

This descriptive cross‐sectional study was conducted in four public hospitals supervised by Kerman University of Medical Sciences. These hospitals were specifically selected because they represent the main tertiary referral and teaching centers in the region, serving a large and diverse patient population and employing nurses across a wide range of clinical wards. Therefore, they provide an appropriate and representative context for examining workplace challenges such as cyberbullying and professional quality of life among nurses. The stages of the present research, from proposal writing to sampling, took place from May 22 to September 29, 2025.

### 2.3. Sampling

All nurses working in educational hospitals affiliated with Kerman University of Medical Sciences were considered the statistical population. In 2025, the total number of nurses across Shafa, Bahonar, Shahid Beheshti, and Afzalipour hospitals was estimated at around 1700. A total sample size of 380 was derived using the Cochran formula (*z* = 1.96, *p* = *q* = 0.5), and to enhance the statistical power of the study, a total of 400 participants were considered. Of these, 335 completed questionnaires were returned and included in the final analysis, resulting in a response rate of 83.75%. Given the use of conservative assumptions in the initial calculation, the final sample size was considered adequate to ensure sufficient statistical power for the analyses performed. Although convenience sampling may limit the generalizability of the findings, this method was considered the most feasible approach given the logistical constraints of the clinical setting, including nurses’ demanding workload and the need to ensure voluntary participation without disrupting patient care activities. Inclusion criteria were at least 6 months of work experience and at least a bachelor’s degree in nursing. Exclusion criteria included incomplete questionnaires.

### 2.4. Study Instruments

The data collection instrument comprised three sections, the first being a researcher‐developed demographic survey assessing age, gender, marital status, education, work experience, ward, shift type, and income.

The second part used a 10‐item cyberbullying questionnaire (unidimensional) to measure cyber harassment. This tool measures individuals’ experiences regarding cyberbullying victimization (such as receiving offensive messages, unpleasant images, or facing online rumors) over the past 2 months. Scoring of the tool is based on a five‐point Likert scale from 1 (never) to 5 (several times a week) [34]. The tool’s reliability was confirmed through internal consistency (Cronbach’s alpha = 0.84), and its content validity was confirmed through the opinions of 5 psychology experts (agreement coefficient = 0.87) [35].

The third part was the “Professional Quality of Life Scale” (ProQOL), which is a standard tool for measuring various dimensions of quality of life in care‐based professions. This tool consists of 25 items measuring two main domains: “CS” (10 items) and “CF” (15 items). The CF dimension comprises two subcomponents: “STS” and “burnout,” each comprising 10 and 5 items, respectively. In interpreting the results, high scores on the occupational fatigue subscale indicate greater vulnerability and risk of developing compassion‐related fatigue; in contrast, high scores on the CS dimension reflect the individual’s sense of effectiveness and gratification from the way care services are provided. Based on the scoring system, the classification of levels in the CS dimension is as follows: scores above 45 indicate a high level, scores below 32.6 indicate a low level, and scores between 32.6 and 45 indicate a moderate level. In the burnout dimension, a score of more than 18.16 indicates severe burnout, a score of less than 8.74 indicates low burnout, and scores between 18.16 and 8.74 indicate a moderate level. In the STS dimension, scores above 32.72 are in the high category, scores below 21.52 are in the low category, and scores between 21.52 and 33.72 are in the moderate category [18]. The 25‐item version of the instrument has been culturally adapted and psychometrically validated in Iran, with acceptable levels of validity and reliability; thus, the Cronbach’s alpha coefficient for the whole tool was reported as 0.73, and that for the three dimensions of CS, STS, and burnout was reported as 0.87, 0.74, and 0.87, respectively [36].

### 2.5. Data Collection Procedure

In the implementation phase, after coordination with the responsible authorities in the target hospitals and visits to various departments, the questionnaires, along with the informed consent form, were provided to eligible nurses for study entry. Sufficient explanations regarding the completion method were provided, and participants were assured that they could answer the questions during their rest time, away from work pressure. In total, 400 paper questionnaires were distributed in this process.

### 2.6. Ethical Consideration

After writing the proposal to receive approval and an ethics code for the present project, it was sent to the Ethics Committee of Kerman University of Medical Sciences, and the code number IR.KMU.REC.1404.139 was received. Participants were fully briefed on the research aims and assured of confidentiality before data collection ensued. Written informed consent was obtained from every individual, and the questionnaires were designed and completed anonymously to ensure the confidentiality of the information received. This study was conducted in accordance with the ethical principles of the Declaration of Helsinki.

### 2.7. Statistical Analysis

Statistical processing was carried out using SPSS Version 24. Descriptive statistics, such as mean, standard deviation, frequency, and percentage, were applied to outline sample characteristics and key variables. Given the data’s normality based on the Kolmogorov–Smirnov test in conjunction with visual inspection of Q–Q plots, independent *t*‐tests and one‐way ANOVA were utilized to analyze demographic variations in CS, burnout, and STS. Given the nonnormal distribution of cyberbullying scores, Spearman’s rank‐order correlation coefficient (Spearman’s rho) was used to assess correlations among the study variables. To identify predictors of the outcome variables, stepwise multivariate regression analyses were conducted. Independent variables for these models were selected based on preliminary bivariate analyses. For each regression model, the statistical assumptions were rigorously verified, including univariate normality, multicollinearity using variance inflation factors and tolerances, and independence of errors via the Durbin–Watson test. Additionally, multivariate outliers were assessed separately for each regression model using Mahalanobis distance statistics. As a result, 40 cases were identified and excluded from the CS and burnout regression models, while 31 cases were excluded from the STS regression model. These exclusions were applied only to the respective regression analyses. All descriptive, bivariate, and correlational analyses were conducted using the full sample of 335 participants. Statistical significance was established at a level of less than 0.05.

## 3. Results

Characteristics of the participants (demographics): The mean age was 32.89 ± 7.66 (Min = 22, Max = 56). The mean years of working experience was 8.99 ± 7.09. The majority of participants were female (66.3%), married (60.3%), and had a bachelor’s degree (83.3%). Most participants had rotated shifts (95.2%) and were working in ICUs (46.0%) (Table 1).

**TABLE 1:** The participants’ characteristics and their association with compassion satisfaction, burnout, and secondary traumatic stress (*n* = 335).

Variable	N (%)	Compassion satisfaction		Burnout		Secondary traumatic stress	
		Mean (SD)	Statistical analysis (*p* value)	Mean (SD)	Statistical analysis (*p* value)	Mean (SD)	Statistical analysis (*p* value)
Age (yr.)							
22–30	165 (49.3)	34.69 (5.31)	*F* = 3.751 (0.024)	15.85 (3.97)	*F* = 2.621 (0.074)	31.38 (5.28)	*F* = 0.537 (0.585)
31–40	115 (34.3)	33.95 (5.50)		16.64 (3.69)		31.75 (4.53)	
> 40	55 (16.4)	36.38 (5.61)		15.27 (4.13)		32.13 (4.46)	

Gender							
Female	222 (66.3)	35.28 (5.59)	*t* = 2.702 (0.007)	16.24 (3.95)	*t* = 1.398 (0.163)	32.14 (4.86)	*t* = 2.711 (0.007)
Male	113 (33.7)	33.59 (5.06)		15.61 (3.85)		30.62 (4.84)	

Marital status							
Single	133 (39.7)	34.42 (5.60)	*t* = −0.794 (0.428)	15.73 (3.80)	*t* = −1.138 (0.256)	30.94 (5.11)	*t* = −2.093 (0.037)
Married	202 (60.3)	34.91 (5.38)		16.23 (3.99)		32.08 (4.71)	

Degree							
B.Sc.	279 (83.3)	35.12 (5.58)	*t* = 3.063 (0.002)	15.87 (4.13)	*t* = −1.621 (0.106)	31.59 (5.15)	*t* = −0.325 (0.745)
M.Sc.	56 (16.7)	32.70 (4.40)		16.80 (2.55)		31.82 (3.40)	

Shift type							
Rotation	319 (95.2)	34.53 (5.42)	*t* = −2.821 (0.005)	16.10 (3.92)	*t* = 1.471 (0.142)	31.64 (4.93)	*t* = 0.263 (0.793)
Fixed	16 (4.8)	38.44 (5.21)		14.62 (3.74)		31.31 (4.38)	

Working experience (yr.)							
≤ 5	143 (42.7)	34.38 (5.09)	*F* = 2.596 (0.052)	16.04 (3.80)	*F* = 1.939 (0.123)	31.50 (5.10)	*F* = 0.100 (0.960)
6–10	85 (25.4)	34.82 (6.18)		16.48 (4.28)		31.73 (5.21)	
11–15	42 (12.5)	33.38 (4.70)		16.57 (3.51)		31.93 (4.01)	
> 15	65 (19.4)	36.17 (5.54)		15.08 (3.87)		31.58 (4.63)	

Ward							
ICU	154 (46.0)	35.47 (5.50)	*F* = 2.253 (0.063)	15.62 (4.28)	*F* = 1.245 (0.292)	31.21 (5.39)	*F* = 0.641 (0.633)
CCU	37 (11.0)	35.00 (3.69)		16.46 (2.62)		32.30 (3.70)	
Emergency	35 (10.5)	33.11 (5.59)		16.91 (4.29)		31.86 (4.45)	
Medical	67 (20.0)	33.60 (5.84)		16.46 (3.50)		32.12 (4.77)	
Surgical	42 (12.5)	34.76 (5.61)		15.71 (3.78)		31.60 (4.53)	

Employment status							
Through a company	75 (22.4)	32.79 (4.93)	*F* = 6.195 (0.002)	16.59 (3.22)	*F* = 2.108 (0.123)	31.52 (4.64)	*F* = 0.496 (0.610)
Employed	184 (54.9)	35.24 (5.40)		16.10 (4.06)		31.85 (4.75)	
Committed to service	76 (22.7)	35.33 (5.75)		15.30 (4.15)		31.20 (4.90)	

*Note:* F: analysis of variance; *t*: independent *t* test.

Abbreviation: SD, standard deviation.

Professional quality of life and cyberbullying: The mean score of CS was 34.71 ± 5.47 (Min = 19 and Max = 50), and the mean scores of burnout and STS were 16.03 ± 3.92 (Min = 5 and Max = 25) and 31.63 ± 4.90 (Min = 19 and Max = 48), respectively. The majority of participants had moderate levels of CS (56.1%, *n* = 188), burnout (71.6%, *n* = 240), and STS (62.7%, *n* = 210). In addition, 40.6% (*n* = 136) of the participants had a low level and 3.3% (*n* = 11) had a high level of CS; 4.8% (*n* = 16) of the participants had a low level and 23.6% (*n* = 79) had a high level of burnout; 2.4% (*n* = 8) of the participants had a low level and 34.9% (*n* = 117) had a high level of STS. The mean score of cyberbullying was 12.11 ± 5.69 (range: 10–50; observed Min = 10 and Max = 41), which is much lower than the midpoint of the questionnaire, i.e., 30. The majority of participants reported low frequencies of cyberbullying experiences across most questionnaire items. Among the reported behaviors, receiving private images, photos, or video clips from colleagues showed the highest frequency and mean score, indicating that this was the most commonly experienced form of cyberbullying in the workplace (Table 2).

**TABLE 2:** Frequency distribution of cyberbullying questionnaire items.

Item	Mean (SD)	Frequency: *n* (percentage)				
		Never	Once or twice a month	Two to three times a month	Once a week	Several times a week
Receiving harsh and offensive electronic text messages from colleagues.	1.34 (0.92)	281 (83.9)	26 (7.8)	7 (2/1)	10 (3)	11 (3/3)
Receiving violent images, photos, or video clips from colleagues via mobile phone.	1.22 (0.76)	303 (90.4)	10 (3)	7 (2/1)	10 (3)	5 (1/5)
Receiving private and intimate images, photos, or video clips from colleagues via mobile phone.	1.43 (0.95)	261 (77.9)	35 (10/4)	15 (4/5)	16 (4/8)	8 (2/4)
Receiving prank phone calls in which the caller remains silent.	1.20 (0.66)	296 (88.4)	21 (6/3)	9 (2/7)	6 (1/8)	3 (0/9)
Receiving rude e‐mails from people in the workplace.	1.14 (0.60)	312 (93.1)	7 (2/1)	9 (2/7)	4 (1/2)	3 (0/9)
Posting insulting words or statements on websites that you browse.	1.14 (0.61)	312 (93.1)	7 (2/1)	7 (2/1)	7 (2/1)	2 (0/6)
Receiving repeated insulting messages from colleagues.	1.15 (0.60)	309 (92.2)	11 (3/3)	6 (1/8)	7 (2/1)	2 (0/6)
Receiving insulting messages from colleagues through chat rooms.	1.15 (0.59)	308 (91.9)	11 (3/3)	9 (2/7)	5 (1/5)	2 (0/6)
Posting insulting words or statements on blogs that you visit.	1.14 (0.66)	315 (94)	6 (1/8)	4 (1/2)	4 (1/2)	6 (1/8)
Colleagues posting unpleasant images or photos on websites that you browse.	1.15 (0.55)	304 (90.7)	18 (5/4)	8 (2/4)	3 (0/9)	2 (0/6)

Abbreviations: *n*, number; SD, standard deviation.

Correlation between cyberbullying and professional quality of life: Bivariate analysis showed that cyberbullying had a significant positive correlation with burnout (*ρ* = 0.120, *p* = 0.029), STS (*ρ* = 0.210, *p* < 0.001), and CF (*ρ* = 0.185, *p* < 0.001). However, no significant correlation was found between cyberbullying and CS (*p* = 0.215). These findings suggest that greater exposure to workplace cyberbullying was associated with higher levels of burnout and STS among nurses (Table 3).

**TABLE 3:** The correlation between professional quality of life dimensions and cyberbullying (*n* = 335).

Variable	Cyberbullying	
	*ρ*	*p* value
Compassion satisfaction	0.068	0.215
Burnout	0.12	0.029
Secondary traumatic stress	0.21	< 0.001
Compassion fatigue	0.185	< 0.001

*Note: ρ*: Spearman’s correlation coefficient (rho).

Correlation between cyberbullying, professional quality of life, and demographics: Bivariate analysis showed that the CS score was significantly associated with age, gender, degree, shift type, and employment status (*p* < 0.05). The oldest group (> 40 years) reported the highest satisfaction (36.38 ± 5.61), followed by the youngest group (22–30 years) at 34.69 ± 5.31, and the lowest satisfaction was observed in the intermediate group (31–40 years) with a mean of 33.95 ± 5.50. Female nurses reported significantly higher CS (35.28 ± 5.59) compared to male nurses (33.59 ± 5.52). Nurses with a B.Sc. degree reported higher satisfaction (35.12 ± 5.58) compared to their M.Sc. counterparts (32.70 ± 4.40). Nurses on fixed shifts reported remarkably higher satisfaction (38.44 ± 5.21) compared to those on rotation (34.53 ± 5.42). Interestingly, nurses committed to service reported the highest satisfaction (35.33 ± 5.75), followed by employed nurses (35.24 ± 5.40). The company nurses reported the lowest scores (32.79 ± 4.93). The burnout score was not associated with any of the demographic or occupational characteristics (*p* > 0.05). In addition, STS had a significant association with gender and marital status (*p* < 0.05). Female nurses reported significantly higher STS scores (32.14 ± 4.86) than male nurses (30.62 ± 4.84), with a *t*‐test significance of *p* = 0.007. Married nurses reported higher STS (32.08 ± 4.71) compared to single nurses (30.94 ± 5.11) (Table 1).

To determine the predictive factors for CS, burnout, and STS, a stepwise multiple regression was performed. For the CS model, the final regression equation was statistically significant and accounted for 8.7% of the variance (Adjusted *R* ^2^ = 0.087, *F* = 7.347, *p* < 0.001). Higher CS was significantly predicted by having a B.Sc. degree rather than a M.Sc., working fixed shifts, having higher employment stability, female gender, and experiencing higher levels of cyberbullying. For the burnout model, the variables explained 3.7% of the total variance (Adjusted *R* ^2^ = 0.037, *F* = 4.301, *p* = 0.014). Higher exposure to cyberbullying and company employment status were significant predictors of increased burnout scores. For the STS model, the regression analysis accounted for 6.5% of the total variance (Adjusted *R* ^2^ = 0.065, *F* = 8.736, *p* < 0.001). Higher levels of STS were significantly predicted by greater cyberbullying exposure, being married, and female gender (Table 4).

**TABLE 4:** Regression coefficients of the effect of underlying variables on compassion satisfaction, burnout, and secondary traumatic stress scores.

Dependent variable	Independent variable	b	S.E	*β*	t	*p* value	Confidence interval of 95%
Compassion satisfaction^∗^	Constant	33.171	2.782		11.923	< 0.001	27.698–38.644
	Degree	−1.927	0.774	−0.132	−2.489	0.013	−3.450–−0.404
	Cyberbullying	0.135	0.050	0.141	2.679	0.008	0.036–0.235
	Shift type	3.506	1.354	0.137	2.590	0.010	0.843–6.169
	Employment status	1.035	0.430	0.127	2.405	0.017	0.189–1.882
	Sex	−1.245	0.611	−0.108	−2.036	0.043	−2.447–−0.042

Burnout^∗∗^	Constant	16.459	0.781		21.067	< 0.001	14.923–17.996
	Cyberbullying	0.079	0.037	0.115	2.4	0.035	0.006–0.153
	Employment status	−0.693	0.317	−0.119	−2.186	0.030	−1.316–−0.069

Secondary traumatic stress^∗∗∗^	Constant	29.196	1.374		21.246	< 0.001	26.492–31.899
	Cyberbullying	0.175	0.046	0.203	3.803	< 0.001	0.084–0.265
	Marital status	1.285	0.533	0.129	2.411	0.016	0.237–0.2.333
	Sex	−1.302	0.555	−0.126	−2.368	0.018	−2.383–−0.221

*Note:* For compassion satisfaction and burnout models, *n* = 295; for secondary traumatic stress model, *n* = 304.

^∗^Adjusted *R* ^2^ = 0.087, *F* = 7.347 (*p* < 0.001).

^∗∗^Adjusted *R* ^2^ = 0.037, *F* = 4.301 (*p* = 0.014).

^∗∗∗^Adjusted *R* ^2^ = 0.065, *F* = 8.736 (*p* < 0.001).

## 4. Discussion

The results indicated that the participating nurses exhibited moderate levels across all three domains of professional quality of life: CS, burnout, and STS. At the same time, the level of experienced cyberbullying in the workplace is relatively low. This pattern is aligned with international evidence showing that a significant portion of the world’s nurses are at moderate levels of CS, burnout, and STS [37]. A systematic review study on cyberbullying among health workers shows that this phenomenon can have consequences such as severe stress, burnout, intention to leave, and decline in care quality. Therefore, it should be considered alongside classic occupational stress factors in nursing [11].

ProQOL dimensions: In the present study, the mean CS score falls in the moderate range. This finding is consistent with Bahari et al.’s study on Saudi nurses, in which most nurses reported moderate levels of CS, with the mean CS score in the middle of the scale [38]. Also, a study conducted in India reported that the majority of nurses experienced moderate levels of CS [29], suggesting that Iranian nurses’ status in this regard is at the global average. Another study on ICU nurses in China reported the mean CS in the moderate range, which is numerically very close to the mean observed in the present study, and this convergence confirms the relative stability of the CS construct in ICUs across different cultures [37]. Data from a study in South Africa showed that ICU nurses predominantly (45%) experience moderate CS [39]. At the same time, some studies, such as a study in Thailand on ICU nurses, reported the mean CS in the moderate range but slightly higher, which may reflect higher psychological capital, resilience‐strengthening interventions, or differences in organizational structure and indicates that the work environment can significantly influence the level of CS [40]. In regional countries, a study in Saudi Arabia reported a slightly higher level of CS than our study results, in the moderate range [41]. Also, CS among Iranian nurses was reported as moderate by most participants in a study [42].

Regarding burnout, about three‐quarters of participants reported a moderate level, and one‐fifth reported a high level. Dixit et al. in India, at the peak of the pressures resulting from COVID‐19, reported moderate burnout in 66% of nurses [29], which is consistent in prevalence with the present study and is concerning. The general pattern of burnout distribution in the present study is similar to the findings of Bahari et al., in which about two‐thirds of nurses had moderate burnout, and a significant proportion were in the high burnout category [38]. The burnout rate among nurses in two studies in Iran and Saudi Arabia was reported in the moderate range and slightly lower than our data [41, 42].

In this investigation, 97% of the nursing staff indicated experiencing moderate to high STS, which indicates significant exposure of nurses to emotional consequences and frequent contact with suffering and trauma. This finding is consistent with the study by Bahari et al., which reported a significant prevalence of STS among nurses. However, the reported value in our study is slightly higher [38]. Also, in a study in Iran, the level of STS was reported in the moderate range, but lower than our data [42]. In a study in India, most nurses had a moderate level of STS [29]. A study in South Africa showed that ICU nurses with a high volume of critically ill patients are at a higher risk of secondary stress [39]; this finding also matches the relatively high mean of STS in the present study, as the main part of the sample in the present study consists of ICU nurses. However, the report of STS in Saudi Arabian nurses was significantly lower than our results [41]. Furthermore, regional research in the Middle East, including studies by Ayed et al. and Batran et al. in Palestine, has shown that the professional quality of life of nurses in crisis‐stricken contexts, especially in terms of secondary stress, is more vulnerable, which could contribute to the similarity of STS levels in the present study with data from those countries [30, 31]. These findings may indicate that nurses working in environments characterized by ongoing stressors and resource constraints are more vulnerable to emotional exhaustion and secondary stress. The convergence of these findings suggests that regional factors—such as high patient acuity, workforce shortages, and socioeconomic pressures—may amplify nurses’ psychological burden.

Cyberbullying: The mean score of cyberbullying in the present study was substantially below the questionnaire’s midpoint. This finding demonstrates a low overall prevalence and a lower frequency of cyberbullying exposure among the surveyed nurses, with the majority of participants reporting minimal experiences of digital harassment across most items. In a systematic review conducted in 2025 within care environments, the prevalence of cyberbullying victimization ranged widely between 1.5% and 46.6%, with cyber incivility among nurses estimated at 36.8% [11], which shows that the findings of the present study are at the lower end of this global spectrum. Conversely, in a study on Korean nurses, a significant proportion reported encountering humiliating messages or exclusion in group chats within a 6‐month period [10], while our sample exhibited a much lower overall frequency. Although the current level of cyberbullying was low in the present study, previous research suggests that even infrequent exposure to workplace cyberbullying may have cumulative negative effects over time. A systematic review of 52 studies reported associations between workplace cyberbullying and adverse outcomes such as psychological distress, burnout, reduced job satisfaction, and increased turnover intentions [17]. In a study in Jordan, the prevalence of cyber victimization among nursing students was reported as 20.4% [13]. Also, another study reported the occurrence of virtual violence in about 19% of nursing students as high or moderate. It showed that cyberbullying and online violence can shape the future behavioral pattern of these new entrants to the health system [8]. Therefore, this highlights the importance of early monitoring and organizational vigilance to prevent low‐level digital incivility from developing into more persistent forms of occupational stress over time.

Correlation of cyberbullying and ProQOL dimensions: In the correlation analysis of the variables in the present study, cyberbullying had a significant positive correlation with STS, burnout, and CF, whereas its direct correlation with CS did not reach statistical significance. This indicates that as electronic harassment increases, nurses exhibit a concurrent increase in the negative dimensions of professional quality of life. The present findings are generally consistent with a 2023 study conducted in China involving 297 nurses. That study showed that workplace bullying is positively associated with STS and burnout [43]. Also, a systematic review on occupational bullying among nurses mentioned that various forms of workplace abuse are heavily tied to elevated burnout and CF [4]. Interestingly, when transitioning to multivariate regression analysis to control for confounding demographic and occupational factors, cyberbullying emerged as a significant positive predictor across all three domains: burnout, STS, and CS. Although cyberbullying was not significantly associated with CS in the bivariate analysis, it emerged as a positive predictor of CS in the adjusted regression model. This difference may be explained by the inclusion of additional demographic and occupational variables in the multivariable analysis, which may have accounted for potential confounding effects and altered the observed association. Therefore, the regression coefficient reflects an adjusted relationship rather than the crude association observed in the bivariate analysis. Given the absence of a significant bivariate association and the relatively low explanatory power of the model, this finding should be interpreted with caution, and further longitudinal and analytical studies are recommended to clarify the direction and underlying mechanisms of the relationship between cyberbullying and CS.

Correlation of ProQOL dimensions with demographic characteristics: The current findings demonstrated that CS was significantly associated with the participants’ age. This pattern closely aligns with a robust body of empirical literature indicating that older, more experienced nurses frequently report higher levels of CS alongside diminished burnout [30, 38]. Furthermore, nurses holding a B.Sc. degree and working fixed shifts exhibited greater CS compared to those with an M.Sc. degree or rotating schedules. Also, shift type and educational level emerged as significant predictors of CS. The lower satisfaction among rotating‐shift nurses is well documented; irregular schedules severely disrupt circadian rhythms and impair work–life balance, thereby progressively eroding professional well‐being [44]. Another notable finding was that nurses with service‐committed employment status reported lower burnout and higher CS than company‐employed nurses, with employment status significantly predicting both subscales. These differences may reflect disparities in job security and organizational support, as company‐employed nurses are more likely to experience unstable working conditions, limited benefits, and lower occupational stability, all of which are associated with increased emotional exhaustion and poorer professional quality of life [45].

In terms of gender, the present study showed that female nurses reported higher levels of CS and STS than their male colleagues. However, no significant difference was observed in terms of burnout. In a study in Turkey, the burnout rate among women was higher than among men [46]. This pattern is consistent with some regional studies, which have reported that female nurses exhibit higher empathy and greater patient engagement, which may increase their exposure to emotional strain while simultaneously enhancing satisfaction derived from the caregiving role [42]. A study conducted in Taiwan on factors associated with nurses’ professional quality of life similarly reported that gender and age were significantly associated with ProQOL subscales and job satisfaction, with female nurses demonstrating higher levels of CS [47]. Married participants reported higher levels of STS than single participants, and marital status was identified as a significant predictor of this subscale. This finding may be explained by the additional emotional and family‐related responsibilities experienced by married nurses, which could increase susceptibility to work‐related psychological stress. Consistent with previous research, family‐related responsibilities, including childcare, may adversely affect nurses’ professional quality of life and contribute to higher levels of STS [38].

## 5. Limitations

Several important methodological limitations must be acknowledged when interpreting the findings of this study. First, the cross‐sectional design precludes the establishment of causal relationships; consequently, it cannot be determined with certainty whether cyberbullying contributes to increased CF or whether the relationship operates in the opposite direction. Second, the reliance on self‐report instruments introduces the inherent risk of social desirability bias and common‐method variance. Finally, the use of a convenience sampling technique limits the generalizability of the results to the broader nursing population.

Conclusion and implication for practice: The findings of the present study, alongside existing global and regional literature, provide a concerning view of moderate to high levels of burnout and STS, with a low level of cyberbullying among nurses, and show that the professional quality of life of this group is under threat. More importantly, this research showed a positive prediction of CF by cyberbullying. This highlights that even low‐intensity or low‐frequency digital incivility carries considerable psychological weight and can meaningfully alter nurses’ occupational well‐being. Consequently, healthcare administrators and policymakers should not overlook low baseline scores of digital harassments; rather, there is a clear imperative to implement proactive organizational policies, cyberethics protocols, and digital support frameworks to safeguard the professional quality of life of the nursing workforce. Therefore, addressing this destructive behavior is necessary to maintain and sustain the nursing workforce in the Iranian health and treatment system.

Systematic reviews on cyberbullying have reported associations between workplace cyberbullying and several unfavorable occupational outcomes, including lower work commitment, sleep disturbances, and turnover intention. In addition, individuals exposed to bullying tend to report a greater likelihood of leaving their jobs. Previous studies have also identified workplace spirituality, organizational support, and resilience as factors associated with better professional quality of life and lower levels of occupational stress. These findings may provide useful considerations for future workforce‐support strategies within the Iranian healthcare system. Also, to address the gaps identified in this study, future research should prioritize several key areas. First, longitudinal studies are required to move beyond associational findings and establish definitive causal pathways between cyberbullying and professional quality of life. Second, interventional research is needed to evaluate the efficacy of empowerment courses like resilience and coping programs and the impact of specific organizational policies on reducing workplace digital violence. Finally, conducting qualitative studies would provide a deeper understanding of the lived experiences and psychological impact of cyberbullying from the perspective of nurses, offering context that quantitative measures may overlook.

At the policy level, current findings indicate that cyberbullying should be placed on the agenda of health managers as an emerging occupational hazard. Structured media literacy, training programs like standardized digital communication protocols, clear guidelines for professional online conduct, and confidential, accessible reporting systems to facilitate safe disclosure and ensure timely organizational response and resilience‐based interventions for nurses and unit managers should be designed and implemented. Also, integrating digital ethics education—incorporating cyberbullying awareness—and communication skills into nursing education programs and in‐service training, especially for young nurses and nursing students, can be effective in reducing the normalization of cyberbullying and promoting a culture of mutual respect.

## Author Contributions

Matin Arabpoor, Fatemeh Malekghasemi, Sara Mehdizadeh, and Morteza Rabiei Vaziri collected the study data. Mahlagha Dehghan performed statistical analyses. Parvin Mangolian Shahrbabaki, Matin Arabpoor, and Fatemeh Malekghasemi wrote the article. Parvin Mangolian Shahrbabaki read the article and made the final necessary checks for its correction.

## Funding

The authors did not receive any financial support for the research, writing, and/or publication of this article.

## Disclosure

All authors approved the article.

## Conflicts of Interest

The authors declare no conflicts of interest.

## Data Availability

All data generated or analyzed during this study are included in this published article.
